# Dual Inhibition of Phosphodiesterase and Ca^++^ Channels Explains the Medicinal Use of *Balanites aegyptiaca* (L.) in Hyperactive Gut Disorders

**DOI:** 10.3390/plants11091183

**Published:** 2022-04-27

**Authors:** Najeeb Ur Rehman, Mohd Nazam Ansari, Wasim Ahmad, Syed Rizwan Ahamad

**Affiliations:** 1 Department of Pharmacology & Toxicology, College of Pharmacy, Prince Sattam Bin Abdulaziz University, Al-Kharj 11942, Saudi Arabia; 2 Department of Pharmacy, Mohammed Al-Mana College for Medical Sciences, Dammam 34222, Saudi Arabia; wasima@machs.edu.sa; 3 Central Laboratory, Department of Pharmaceutical Chemistry, College of Pharmacy, King Saud University, Riyadh 11451, Saudi Arabia; rizwanhamdard@gmail.com

**Keywords:** *B. aegyptiaca*, antispasmodic, Ca^++^ channel blocker, phosphodiesterase inhibitor

## Abstract

The present study attempted to evaluate and rationalize the medicinal use of the methanolic extract of the fruits of *Balanites aegyptiaca* (*B. aegyptiaca*) in the treatment of hyperactive gut disorders. The in vivo, castor oil-induced diarrhea model in mice was followed to test its antidiarrheal effect. To test the antispasmodic effect and to explore its pharmacodynamic details, isolated small intestines (ileum) obtained from rats were selected to provide physiological conditions for the ex vivo assays. In the in vivo assays, the orally administered extract of *B. aegyptiaca* protected mice from diarrheal drops with resultant percent inhibitions of 40% and 80% at the respective doses of 200 mg/kg and 400 mg/kg, while the highest protection (100%) was observed with a positive control drug, loperamide, at 10 mg/kg. In the ileum, *B. aegyptiaca* produced an antispasmodic effect in a concentration-dependent manner by inhibiting the carbachol (CCh; 1 µM) and high K^+^ (80 mM)-evoked spasms with resultant EC_50_ values of 1.44 mg/mL (1.08–1.78) and 1.27 mg/mL (0.98–1.66), respectively. Papaverine, a known phosphodiesterase enzyme (PDE) inhibitor and blocker of Ca^++^ channels (CCB), also inhibited both CCh and high K^+^ induced contractions at comparable EC_50_ values of 8.72 µM (7.92–9.24) and 8.14 µM (7.62–8.84), respectively. Contrary to the extract and papaverine, verapamil showed distinctly higher potency in regard to inhibiting high K^+^, compared to CCh-evoked spasms that had EC_50_ values of 0.16 µM (0.13–0.261) and 2.54 µM (2.28–2.92), respectively. The inhibitory effects of *B. aegyptiaca* on PDE were further confirmed when the pre-incubated extract shifted the isoprenaline-mediated relaxation curves (CRCs) towards the left, similar to papaverine, whereas the CCB-like effect was confirmed when the pre-incubated tissues with *B. aegyptiaca* caused deflection in the Ca^++^ CRCs towards the right, constructed in Ca^++^ free medium with suppression of the maximum response. Thus, this study provides detailed, mechanistic support for the medicinal use of *B. aegyptiaca* in the treatment of hyperactive gut disorders.

## 1. Introduction

Hyperactive gastrointestinal disorders, including diarrhea, are known to contribute significantly to morbidity and mortality in the general population [1,2]. In developed countries, the prevalence of diarrhea is estimated to be 1–5% of the adult population [3]. By definition, diarrhea occurs when a hyper bowel movement causes body water and electrolyte loss, resulting in abnormal stool consistency and a defecation frequency that is three or more times faster than usual with abnormal stool consistency over 24 h [4]. The available therapeutic options to treat diarrhea include both pharmacological and non-pharmacological interventions that aim to reduce the dehydration and discomfort caused by frequent bowel movements [5,6]. Probiotics, enkephalinase inhibitors, bismuth compounds, anticholinergics, opioid agonists, and α2 receptor agonists are some of the most common antisecretory and gut motility suppressants [5,7,8]. These medications have side effects that limit their use, such as dry mouth, distension, abdominal cramps, severe constipation, nausea, and vomiting [9]. Furthermore, respiratory depression and paralytic ileus are two of the most serious side effects associated with opioid agonists such as loperamide [10].

According to a report published by the World Health Organization [1], traditional medicine (TM) is either the core health care delivery pillar in many countries of the world or serves as a complementary treatment. Indeed, since the beginning of mankind, medicinal plants and/or natural products have played a pivotal role in the development of potent therapeutic agents, including antidiarrheal treatments, and about 80% of the population in developing countries still relies on traditional medicine and natural products for their health care [11]. The exploration of herbal products for the treatment of multiple chronic diseases is currently in great demand [12,13]. Medicinal plants with antidiarrheal activity, according to numerous reports on traditional medicine, have fewer side effects than conventional drugs due to the presence of phytoconstituents such as tannins, alkaloids, flavonoids, and terpenoids [14].

**Figure 1:**
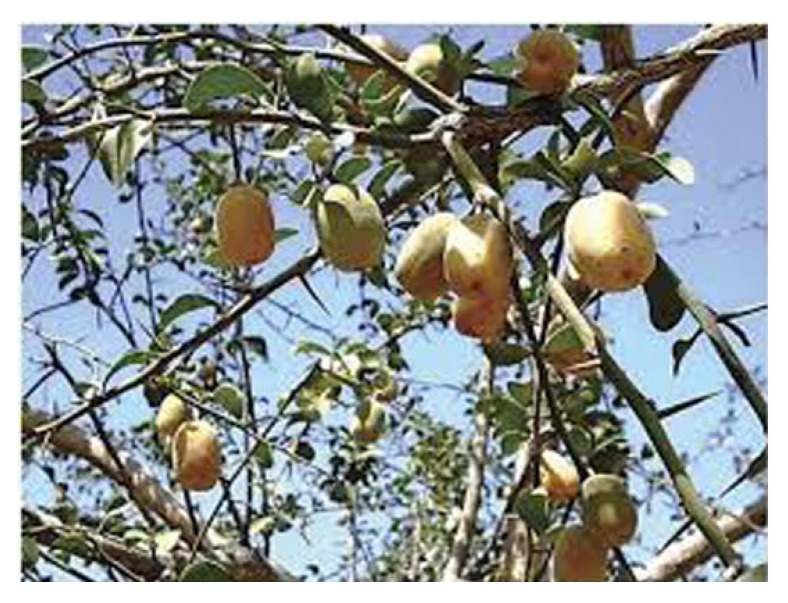
*B. aegyptiaca* L.

*Balanites aegyptiaca* L. Del. (*B. aegyptiaca*) belongs to the family Zygophyllaceae. The word "balanites" was derived from the Greek word "acorm", which means fruit, and was coined in 1813 by Alire Delile, who substituted the Agihalid name, which was derived from the Arabic word "heglig" [15]. The plant is a multibranched, spiny shrub or tree that can grow up to l0 m tall (Figure 1). The fruits are rather long, narrow drupes, around 2.5 to 7 cm long and 1.5 to 4 cm in diameter. The young fruits are green and tomentose, though they turn yellow and glabrous when mature. The pulp is bittersweet and edible. The fruit of *B. aegyptiaca* is commonly known as the desert date in English. *B. aegyptiaca* L. is an important tree in the semiarid ecosystem, and it has beneficial qualities. It is widely distributed in most arid to sub-humid areas of Africa and South Asia [16]. It contains several secondary metabolites, including alkaloids, steroids, flavonoids, phenolics, and tannins [17,18]. It has traditionally been used to treat a wide range of ailments, including malaria, syphilis, helminthic and microbial infections, leukoderma, cancer, jaundice, and diabetes, and it is also a purgative and an emetic agent with high antioxidant potential [19,20,21,22,23,24,25].

According to a literature survey of traditional medicine, *B. aegyptiaca* has been widely used in the treatment of several disorders, but to the best of our knowledge, until today, there has been no detailed study conducted on the therapeutic potential of *B. aegyptiaca* in the treatment of hyperactive gut disorders. Therefore, this study was planned to investigate and discover the exact mechanism(s) implicated in the putative gastrointestinal inhibitory effects of methanolic extract of *B. aegyptiaca* fruits by using in vivo and ex vivo experiments.

## 2. Results

### 2.1. Methanolic Extract Yield (%)

The percentage yield of the finally concentrated, semi-solid fruit part of the methanolic extract of *B. aegyptiaca* was recorded as 25.97%.

### 2.2. Acute Toxicity

The extract of *B. aegyptiaca* was well tolerated by the mice, as no mortality was observed, and no symptoms of toxicity were recorded.

### 2.3. In Vivo Antidiarrheal Effect

Both of the increasing, orally-administered doses of *B. aegyptiaca* in mice showed significant antidiarrheal effects compared to the saline group (Table 1). In mice administered with the lower tested dose of 200 mg/kg, two out of the five cages of mice had no diarrheal spots, thus resulting in an antidiarrheal effect of 40%, while the animals that received the higher dose of 400 mg/kg had diarrheal drops in one cage, thus resulting in an antidiarrheal effect of 80%. In mice that were given an orally-administered dose of 10 mg/kg of loperamide, none of the cages had diarrheal spots, resulting in 100% protection. All the details are shown in Table 1.

### 2.4. Ex Vivo Antispasmodic Effects

**Figure 2:**
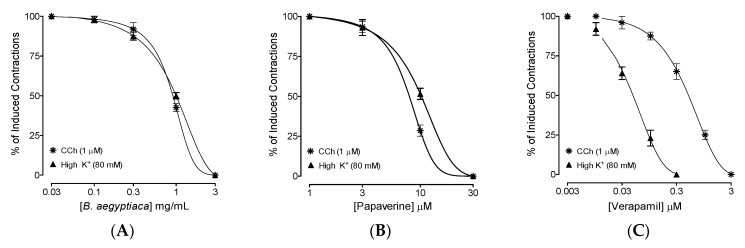
Concentration–response curves showing comparison of the (**A**) methanolic fruits extract of *B. aegyptiaca*, (**B**) papaverine, and (**C**) verapamil for the inhibitory effect against carbachol (CCh; 1 µM) and high K^+^ (80 mM)-induced contractions in isolated rat ileum preparations. Values shown are mean ± SEM (*n* = 4–5).

In the contracted ileum tissues evoked by CCh and high K^+^, the extract of *B. aegyptiaca* initiated its relaxant effect at 0.1 mg/mL and expressed complete inhibition at 3 mg/mL, with resultant EC_50_ recorded as 1.44 mg/mL (1.08–1.78, 95% CI, *n* = 4–5) and 1.27 mg/mL (0.98–1.66, 95% CI, *n* = 4–5), respectively, against CCh and high K^+^ (Figure 2A). A pure drug, papaverine, was used as a positive control and showed similar and comparable relaxant effects against CCh and high K^+^-evoked spasms, with EC_50_ values recorded as 8.72 µM (7.92–9.24, 95% CI, *n* = 4–5) and 8.14 µM (7.62–8.84, 95% CI, *n* = 4–5), (Figure 2B). Contrary to the extract and papaverine, verapamil inhibited high K^+^-evoked sustained contractions in the ileum at significantly lower concentrations compared to its inhibited CCh-evoked contractions, and the resultant EC_50_ values recorded were 0.16 µM (0.13–0.261, 95% CI, *n* = 4–5) and 2.54 µM (2.28–2.92, 95% CI, *n* = 4–5), respectively (Figure 2C).

### 2.5. Phosphodiesterase Enzyme (PDE) Inhibitory-Like Effect

**Figure 3:**
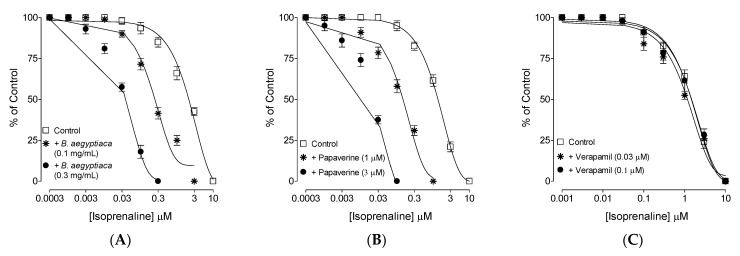
Inhibitory concentration–response curves of isoprenaline against carbachol (CCh)-induced contractions in the absence and presence of different concentrations of (**A**) methanolic fruits extract of *B. aegyptiaca*, (**B**) papaverine, and (**C**) verapamil in isolated rat ileum preparations. Values shown are mean ± SEM (*n* = 4–5).

After assessing the extract’s inhibitory CRCs against CCh and high K^+^ at comparable EC_50_ values similar to that of papaverine (Figure 2), further experiments were conducted to confirm the papaverine-like PDE-inhibitory activity of the plant extract. The ileum tissues that were pretreated with *B. aegyptiaca* at two increasing concentrations of 0.1 and 0.3 mg/mL caused leftward deflection in the isoprenaline-mediated inhibitory CRCs (Figure 3A), thus resulting in potentiation. Papaverine (1 and 3 μM) also caused a similar leftward shift of the CRCs of isoprenaline (Figure 3B), while verapamil did not affect the inhibitory CRCs of isoprenaline (Figure 3C).

### 2.6. Calcium Channel Blocking (CCB)-like Effect

**Figure 4:**
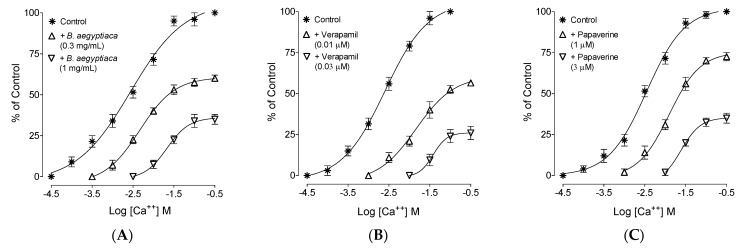
Concentration–response curves of Ca^++^ in the absence and presence of the increasing concentrations of the (**A**) methanolic fruits extract of *B. aegyptiaca*, (**B**) verapamil, and (**C**) papaverine in isolated rat ileum preparations. Values shown are mean ± SEM (*n* = 4–5).

In the earlier observed inhibitory CRCs of *B. aegyptiaca* extract against high K^+^ (Figure 2A), which is an indication of a CCB-like effect, further confirmation was made by constructing and comparing the CRCs of Ca^++^ added exogenously to the tissues that were previously made Ca^++^ free. Ileum tissues that were pre-incubated with *B. aegyptiaca* extract deflected the Ca^++^ CRCs towards the right, with suppression of the maximum contractile response recorded in the absence of the extract (Figure 4A). A similar type of suppression and rightward deflection in the Ca^++^ CRCs was recorded in tissues pre-incubated with verapamil (0.01 and 0.03 µM) and papaverine (1 and 3 µM), as seen in Figure 4B and C, respectively.

## 3. Discussion

The medicinal report of *B. aegyptiaca* in diarrhea and gut spasms [16] was evaluated scientifically and in detail using rodents. The safety evaluation and antidiarrheal effect of the methanolic fruit extract of *B. aegyptiaca* were tested in mice using acute toxicity and a castor-oil-evoked diarrhea model, respectively. The orally administered methanolic fruit extract of *B. aegyptiaca* exhibited no toxic effects at doses up to 4000 mg/kg body weight. The observed normalcy, insignificant behavioral changes, and related parameters reveal the safety of the extract. A previously reported study that was conducted on rats by Hassan et al. [26] also shows the safety of the *B. aegyptiaca* fruit extract, up to a dose of 4000 mg/kg body weight. Similarly, our current findings regarding the safety of the plant are further supported by the acute and sub-acute toxicity study reported by Ali et al. [27], which shows that *B. aegyptiaca* seed oil did not affect the hematological parameters, such as the hemoglobin concentration, packed cell volume, red blood cell (RBC) count, mean corpuscular volume, and mean corpuscular hemoglobin concentration, of Wistar albino rats. Additionally, their study revealed no signs of toxicity in regard to biochemical parameters (alanine aminotransferase (ALT), aspartate aminotransferase (AST), alkaline phosphatase (ALP), total proteins, creatinine, and urea), and recorded no toxicity while analyzing the histopathology of different tissues, such as the liver, lung, heart, kidney, intestine, stomach and spleen. The in vivo tests resulted in a dose-mediated antidiarrheal effect by inhibiting the typical diarrheal drops that were seen in the saline control group. The diarrheagenic effect of castor oil in normal mice is mediated after its hydrolysis to ricinoleic acid, which irritates the intestines and causes the inhibition of water and electrolyte absorption, finally leading to powerfully evoked spams in the gut [28]. *B. aegyptiaca* pre-incubation at two increasing doses protected mice from castor-oil-evoked diarrhea by 40% and 80%, respectively. However, the maximum protection was observed with loperamide, which is a reported antidiarrheal agent [29] and a positive control that was used in this study. Our next aim was to explore the detailed mechanism(s) of action that were responsible for the observed antidiarrheal effect in mice. The methanolic extract of *B. aegyptiaca* was evaluated in cumulative concentrations in isolated rat ileum by following the previously reported assays, which show that antidiarrheal and antispasmodic substances produce gut-relaxant effects via the blockade of Ca^++^ channels [29,30], PDE inhibition, or both [31]. Hence, we investigated *B. aegyptiaca* extract on provoked contractions in rat ileum with CCh and high K^+^ as contractile agents [32], and interestingly, the plant showed comparable inhibitory CRCs against both types of contractile agents with no statistical difference (*p* ˃ 0.05). We compared our findings with the papaverine, a known inhibitor of dual nature against Ca^++^ channels, and PDE [33], which reversed both CCh and high K^+^-evoked spasms at comparable EC_50_ values (*p* < 0.05), whereas verapamil, an inhibitor of Ca^++^ channels [34,35], selectively showed greater potency to inhibit K^+^ compared to CCh-evoked spasms. This shows that *B. aegyptiaca* possibly contains the dual mechanism(s) of PDE inhibition and Ca^++^ channels, similar to papaverine. *B. aegyptiaca* was also tested indirectly for PDE inhibition and cAMP elevation, as PDE inhibitors cause cAMP elevation in tissues by blocking PDE. PDE lowers the cAMP concentration in tissues as it converts cAMP to AMP, which is inactive in nature, and thus, PDE is a hurdle to smooth muscle relaxation [36]. The shifting of isoprenaline’s inhibitory CRCs against CCh towards lower doses (leftward) in pre-incubated ileum tissues of *B. aegyptiaca* indirectly indicates the inhibitory effect of the extract on PDE, and a similar potentiation effect was noticed with papaverine, a standard PDE inhibitor [37]. PDE inhibitors are well known for inhibiting CCh-mediated smooth muscle spasms [38].

A substance that reverses high K^+^ (˃30 mM)-mediated spasms depicts CCBs [39]; hence, to support and further confirm the CCB-like action of *B. aegyptiaca*, the ileal tissues were pre-incubated with *B. aegyptiaca* at increasing concentrations in previously Ca^++^ free tissues. Cumulative contractile Ca^++^ CRCs were achieved in the absence (vehicle control) and in the pre-incubated tissues with *B. aegyptiaca*, where deflection towards the right with suppression of the maximum peak was noticed in Ca^++^ CRCs. Similarly, papaverine, an inhibitor of PDE and Ca^++^ channels (dual nature), and verapamil, a standard CCB, were also reported for similar suppression of Ca^++^ CRCs [34], thus confirming the CCB-like effect of *B. aegyptiaca*.

## 4. Materials and Methods

### 4.1. Extraction of Plant Material

*B. aegyptiaca* fruits were purchased from Seiko market, Dammam (KSA) and authenticated (PL/045/2020-21/P-010) by Department of Pharmacognosy, College of Clinical Pharmacy, Taif University (KSA). The dried, powdered plant material (40 gm) was used for the extraction, along with 200 mL methanol at 70 °C using soxhlet apparatus. After extraction, the extract was dried using rotary evaporator. The dried extract was stored in airtight glass container at 5–10 °C and used for further study. The percentage yield was calculated using the given formula.

$$\%\;\mathrm{Extraction}\;\mathrm{yield}=\frac{\mathrm{Weight}\;\mathrm{of}\;\mathrm{dried}\;\mathrm{extract}}{\mathrm{Weight}\;\mathrm{of}\;\mathrm{drug}\;\mathrm{sample}}\times 100$$

### 4.2. Chemicals

The analytical-grade chemicals used in this study obtained from Sigma Company (St. Louis, MO, USA) include carbachol, loperamide hydrochloride, acetylcholine perchlorate (ACh), isoprenaline, verapamil, and papaverine. The physiological buffer (Tyrode) composition includes different salts obtained from Merck, Germany, and also includes calcium chloride, glucose, magnesium sulfate, potassium chloride, potassium di-hydrogen phosphate, sodium bicarbonate, and sodium chloride. For diarrhea induction, castor oil procured from local pharmacy was used.

### 4.3. Animals

Wistar albino rats (200–230 g) for the ex vivo experiment and Swiss albino mice (30–35 g) for the in vivo study, housed at the animal care facility located in the College of Pharmacy, Prince Sattam bin Abdulaziz University (KSA), were used by following the instructions detailed in NRC [40]. Standard conditions required to house the laboratory animals were provided, such as optimal temperature (23 ± 1 °C), relative humidity (55 ± 5%), and equal light/dark cycle exposure. All animals had free access to a standard pellet diet and water. The conducted research study protocol was assigned an approval number of BERC-004-12-19 by the institutional Bio Ethical Research Committee (BERC).

### 4.4. Acute Toxicity Study

The detailed guidelines reported in OECD-423 were followed to calculate the maximum tolerated dose of *B. aegyptiaca* using an acute toxicity test [41]. Briefly, overnight fasted albino mice (*n* = 6) were distributed into two groups in a random fashion. One group of animals was exposed to oral administration of *B. aegyptiaca* (2 g/kg), while the second group, labeled as a vehicle control group, only received vehicle (0.9% saline) with a dose of 10 mL/kg. Both groups of mice were maintained in similar housing conditions, and each individual rat was continuously observed for 4 h for any sign of toxicity/or death in 15 min intervals, then every half hour for the next 6 h, and then daily for two days (48 h). Since no mortality was observed at 2 g/kg, the dose of *B. aegyptiaca* was increased to 4 g/kg, and the treated mice were further observed for the next 48 h.

### 4.5. In Vivo Antidiarrheal Study

For diarrheal protection assay, a previously reported method [42] was followed, with some modifications. Briefly, twenty mice of either gender were randomly distributed into four groups (*n* = 5). A fasting period of twenty-four hours was followed for all animals, with free access to water. The fasted mice in 1st group were exposed to saline (10 mL/kg) using oral gavage and were labeled negative control group. On the basis of preliminary screening for dose selection of *B. aegyptiaca*, 2nd and 3rd groups (test groups) were orally administered 200 and 400 mg/kg of the *B. aegyptiaca* extract, respectively. The 4th group of mice, labeled as positive control group, was exposed to loperamide (10 mg/kg, orally), a standard antidiarrheal agent. After dosage was administered, each individual mouse was isolated from its group and was placed in a separate cage that was floored with a blotting paper instead of husk for recording any diarrheal spots/drops by a blind observer. One hour after the tested and control drug treatments, each individual mouse was orally administered castor oil (10 mL/kg). After 4 h, each individual cage of mice was opened to observe the typical diarrheal spots/drops on their lined blotting sheets via a blind observation. Individual animal protection was recorded only if no diarrheal drops were observed [42,43].

### 4.6. Ex Vivo Experiments on Isolated Rat Ileum

The method cited in our earlier published article was followed to test the antispasmodic effect of the extract of *B. aegyptiaca* [44]. Adult rats (200–230 g) were sacrificed via cervical dislocation after light anesthesia, and their ilea were carefully isolated and preserved in Tyrode’s solution bubbled with carbogen (95% O_2_ + 5% CO_2_). The required segments of tissues (2–3 cm length) were cleaned off from attached mesenteric tissues via sharp scissors, whereas the inside luminal fecal materials were cleaned by blowing Tyrode’s solution using 5 mL syringe. Each individually prepared ileum tissue was mounted in 20 mL organ bath (emkaBath; France) with inbuilt, fixed transducer and tracings record (IOX software). The tissue was continuously supplied with fresh Tyrode’s medium bubbled with carbogen, whereas temperature was maintained at 37 °C by supplied heater. The millimolar (mM) concentration of the individual slats of the solution was KCl 2.68, NaCl 136.9, MgCl_2_ 1.05, NaHCO_3_ 11.90, NaH_2_PO_4_ 0.42, CaCl_2_ 1.8, and glucose 5.55 (pH 7.4). Initial tension of 1 g was kept and maintained, and each tissue was incubated in 30 min intervals without any drugs except the Tyrode’s solution, which was replaced every 10 min. After 30 min, each individual ileum tissue was exposed to multiple exposures of acetylcholine (0.3 µM) until the same contractile response, called stabilization, was obtained. After stable Ach responses occurred, sustained contractions were evoked using CCh and high K^+^, and the tested sample (*B. aegyptiaca*) was added cumulatively to the bath solution starting from 0.01 mg/mL and increasing until maximum final bath concentration (FBC) of 10 mg/mL was reached. When the inhibitory effect of *B. aegyptiaca* against spasmogens of CCh and K^+^ (80 mM) was achieved, further experiments were conducted to determine the precise pharmacodynamics for the observed antispasmodic effects. Previous reports indicate that substance with antispasmodic potential exerts its effect by antagonizing Ca^++^ channel and/or inhibiting PDE [43,44], in addition to other mechanism(s) [42]. The earlier reported study by Godfraind et al. [31] shows that a tissue exposed to final bath concentration of high potassium [K^+^ (>30 mM)] evoked excitations of multiple smooth muscles by opening L-type Ca^++^ channels, thus resulting in powerful, sustained contractions. Similarly, any substance that produces relaxation of the sustained contraction caused by either CCh and/or high K^+^ at comparable concentrations is categorized as PDE inhibitor [45].

### 4.7. Ca^++^ Inhibitory Confirmation

When *B. aegyptiaca* inhibited the contractions mediated by high K^+^ in the tested dose range, further assays were conducted to confirm the Ca^++^ inhibitory action (CCB) of *B. aegyptiaca* extract following earlier reported methods [33]. Briefly, dose-mediated CCB effect of *B. aegyptiaca* was determined by constructing CRCs of Ca^++^ in ileal tissue previously made Ca^++^ free by incubating the tissues for 45 min in a Ca^++^-free buffer medium with additional mixing of chelating agent, EDTA (0.1 mM), to chelate Ca^++^. To further deplete the intracellular Ca^++^ stores of ileum smooth muscles, the buffer medium was replaced with medium having higher ratio of potassium (K^+^-rich and Ca^++^-free Tyrode solution). The incubated ileum tissue in these buffer media was confirmed as completely Ca^++^-free if it was insensitive to contractile response of Ach (0.3 uM). After the tissue was confirmed to be Ca^++^-free, CaCl2 was added exogenously to the tissue bath to obtain the control Ca^++^ CRCs. The CRCs of Ca^++^ were reproduced in the presence of the pre-incubated ileum tissues with increasing concentrations of *B. aegyptiaca*, and the results were compared with standard CCB agent, verapamil [34].

### 4.8. PDE Inhibitory Confirmation

*B. aegyptiaca*’s relaxation effect against the sustained contraction achieved with CCh and high K^+^ at similar concentrations is indication of its dual inhibition of PDE inhibition and Ca^++^ channels [45,46]; therefore, we used assays to further confirm the PDE-inhibitory potential of *B. aegyptiaca* following a previously reported method [47]. The constructed, cumulative CRCs of isoprenaline against CCh in the presence of vehicle (control) and presence of pre-incubated tissues with increasing concentrations of *B. aegyptiaca* were used to assess PDE inhibition indirectly. The potentiation of the isoprenaline curves towards left indicates PDE inhibition when compared to papaverine, a standard PDE inhibitor [47].

### 4.9. Statistics

The obtained findings were represented as mean standard error of mean (SEM), whereas “*n*” represents the number of repetitions of the individual experiment. The values of median effective concentrations (EC_50_) were analyzed while keeping 95% confidence intervals (CI). The CRCs obtained from ex vivo experiments were compared with their respective controls by applying Student’s *t*-test or two-way ANOVA, followed by Bonferroni’s post-test. Protection from castor-oil-mediated diarrhea was analyzed by comparing all the three treatment groups with normal control group via Chi-square (χ2) test. *p* < 0.05 was considered to be statistically significant. For the regression analysis of CRCs, Graph-Pad prism (Version 4) was used as a statistical application.

## 5. Conclusions

The data observed in the present study suggest that the methanolic extract of *B. aegyptiaca* exhibits antidiarrheal and antispasmodic activities, possibly mediated through combination of PDE-inhibition and Ca^++^ channel antagonist-like mechanisms, though additional mechanism(s) cannot be ignored. Therefore, it is recommended that in the future, *B. aegyptiaca* may be used clinically for the treatment of diarrhea.

## Figures and Tables

**Table 1:** Antidiarrheal activity of the methanolic fruits extract of *B. aegyptiaca* on castor-oil-induced diarrhea in mice.

Treatment (p.o.), Dose (mg/kg)	No. of Mice with Diarrhea	% Protection
Saline + Castor oil 10 (mL/kg) + 10 (mL/kg)	5/5	0
*B. aegyptiaca* + Castor oil 200 (mg/kg) + 10 (mL/kg) 400 (mg/kg) + 10 (mL/kg)	3 */5 1 */5	40 80
Loperamide + Castor oil 10 (mg/kg) + 10 (mL/kg)	0 **/5	100

* *p* < 0.05 and ** *p* < 0.01 vs. Saline + Castor oil treatment group (χ^2^-test).

## Data Availability

Not Applicable.
